# Obesity Does Not Modulate the Glycometabolic Benefit of Insoluble Cereal Fibre in Subjects with Prediabetes—A Stratified Post Hoc Analysis of the Optimal Fibre Trial (OptiFiT)

**DOI:** 10.3390/nu11112726

**Published:** 2019-11-11

**Authors:** Stefan Kabisch, Nina Marie Tosca Meyer, Caroline Honsek, Christiana Gerbracht, Ulrike Dambeck, Margrit Kemper, Martin A. Osterhoff, Andreas L. Birkenfeld, Ayman M. Arafat, Martin O. Weickert, Andreas F.H. Pfeiffer

**Affiliations:** 1 Department of Clinical Nutrition, German Institute of Human Nutrition Potsdam-Rehbrücke, Arthur-Scheunert-Allee 114-116, 14558 Nuthetal, Germany; nina.meyer@dife.de (N.M.T.M.); caroline.honsek@gmail.com (C.H.); Christiana.Gerbracht@dife.de (C.G.); ulrikedambeck@gmx.de (U.D.); margrit.kemper@dife.de (M.K.); martin@cm-osterhoff.de (M.A.O.); Ayman.Arafat@charite.de (A.M.A.);; 2 Deutsches Zentrum für Diabetesforschung e.V., Geschäftsstelle am Helmholtz-Zentrum München, Ingolstädter Landstraße 85764 Neuherberg, Germany; Andreas.Birkenfeld@uniklinikum-dresden.de; 3 Department of Endocrinology, Diabetes and Nutrition, Campus Benjamin Franklin, Charité University Medicine, Hindenburgdamm 30, 12203 Berlin, Germany; 4 Medical Clinic IV, Department of Diabetology, Endocrinology, Nephrology, University Clinic Tübingen, Hoppe-Seyler-Straße 3, 72076 Tübingen, Germany; 5 Institute for Diabetes Research and Metabolic Diseases, Helmholtz Zentrum Munich at the University Tübingen, Ottfried-Müller-Straße 10, 72076 Tübingen, Germany; 6 Warwickshire Institute for the Study of Diabetes, Endocrinology and Metabolism; The Arden Net Centre, Enets CoE, University Hospitals Coventry and Warwickshire NHS Trust, Coventry CV2 2DX, UK; M.weickert@outlook.com; 7 Centre of Applied Biological & Exercise Sciences (ABES), Faculty of Health & Life Sciences, Coventry University, Coventry CV1 5FB, UK; 8 Translational & Experimental Medicine, Division of Biomedical Sciences, Warwick Medical School, University of Warwick, Coventry CV4 7AL, UK

**Keywords:** diabetes mellitus type 2, prediabetes, diabetes prevention, obesity, stratification, impaired glucose tolerance, insoluble dietary fibre, insulin sensitivity

## Abstract

Obesity does not modulate the glycometabolic benefit of insoluble cereal fibre in subjects with prediabetes—a stratified post hoc analysis of the Optimal Fibre Trial (OptiFiT). Background: OptiFiT demonstrated the beneficial effect of insoluble oat fibres on dysglycemia in prediabetes. Recent analyses of OptiFiT and other randomised controlled trials (RCTs) indicated that this effect might be specific for the subgroup of patients with impaired fasting glucose (IFG). As subjects with IFG are more often obese, there is a need to clarify if the effect modulation is actually driven by glycemic state or body mass index (BMI). Aim: We conducted a stratified post hoc analysis of OptiFiT based on the presence or absence of obesity. Methods: 180 Caucasian participants with impaired glucose tolerance (IGT) were randomised in a double-blinded fashion to either twice-a-day fibre or placebo supplementation for 2 years (*n* = 89 and 91, respectively). Once a year, they underwent fasting blood sampling, an oral glucose tolerance test (oGTT) and full anthropometry. At baseline, out of 136 subjects who completed the first year of intervention, 87 (62%) were classified as OBESE (BMI >30) and 49 subjects were NONOBESE. We performed a stratified per-protocol analysis of the primary glycemic and secondary metabolic effects attributable to dietary fibre supplementation after 1 year of intervention. Results: Neither the NONOBESE nor the OBESE subgroup showed significant differences between the respective fibre and placebo groups in metabolic, anthropometric or inflammatory outcomes. None of the four subgroups showed a significant improvement in either fasting glucose or glycated haemoglobin (HbA1c) after 1 year of intervention and only OBESE fibre subjects improved 2 h glucose. Within the NONOBESE stratum, there were no significant differences in the change of primary or secondary metabolic parameters between the fibre and placebo arms. We found a significant interaction effect for leukocyte count (time × supplement × obesity status). Within the OBESE stratum, leukocyte count and gamma-glutamyl transferase (GGT) levels decreased more in the fibre group compared with placebo (adjusted for change in body weight). Comparison of both fibre groups revealed that OBESE subjects had a significantly stronger benefit with respect to leukocyte count and fasting C-peptide levels than NONOBESE participants. Only the effect on leukocyte count survived correction for multiple comparisons. In contrast, under placebo conditions, NONOBESE subjects managed to decrease their body fat content significantly more than OBESE ones. Intention-to-treat (ITT) analysis resulted in similar outcomes. Conclusions: The state of obesity does not relevantly modulate the beneficial effect of cereal fibre on major glycometabolic parameters by fibre supplementation, but leukocyte levels may be affected. Hence, BMI is not a suitable parameter to stratify this cohort with respect to diabetes risk or responsiveness to cereal fibre, but obesity needs to be accounted for when assessing anti-inflammatory effects of fibre treatments. Targeted diabetes prevention should focus on the actual metabolic state rather than on mere obesity.

## 1. Introduction

Worldwide, the type 2 diabetes mellitus (T2DM) epidemic is spreading. As the sedentary lifestyle with cheap and easy access to highly processed food becomes accessible to more and more people, T2DM accounts for a growing number of patients with cardiovascular disease, cancer and other long-term complications, contributing to premature death [1,2,3,4]. T2DM onset and progression can be influenced by eating behaviour, physical activity and other lifestyle factors. Thus, prevention of and therapy for T2DM focus on an optimal diet and reducing sedentary behaviour and other harmful habits. This also includes cessation of smoking [5]. Large prevention trials have shown the potential of lifestyle modification to reduce diabetes incidence by about 40–60% [6,7,8,9].

Major dietary factors promoting the development of T2DM are hypercaloric food intake and resulting obesity, high intake of saturated fat, simple carbohydrates and alcohol. Protective nutritional factors seem to be moderate alcohol intake, coffee consumption and insoluble dietary fibre [10,11,12]. By contrast, soluble fibre—derived from fruits and vegetables—has so far not been shown to counteract diabetes onset [13,14].

Most prevention strategies follow a combined approach, targeting several components at once. However, the actual metabolic potency of the respective single elements is not well investigated. Dietary recommendations are mainly based on observational trials, as randomised controlled trials (RCTs) are often lacking [10,11,12]. With respect to dietary fibre, the Optimal Fibre Trial on Diabetes Prevention (OptiFiT), first published in 2018, was the first large, long-term randomised controlled trial (RCT) specifically targeting dietary insoluble fibre. There are no such RCTs for soluble fibre, and there is no other previous large-scale RCT on insoluble fibre investigating any kind of metabolic or other outcome. OptiFiT has reported significant improvements of 2 h glucose levels (oral glucose tolerance test; oGTT) in women and on glycated haemoglobin (HbA1c) in the entire cohort due to 1 year fibre supplementation [15].

Up to now, there is no proven rationale concerning the mechanism of action of cereal fibre, as studies on possible incretin effects or changes of the gut microbiome have led to inconsistent results. Cereal fibre is mainly composed of cellulose and hemicelluloses, both of which are quite resistant to intestinal fermentation. Thus, they do not relevantly contribute to the production of short-chain fatty acids (SCFA). In contrast, fibre-rich whole-grain products have been shown to increase the production of SCFA and the secretion of glucagon-like peptide (GLP-1) [16]. The authors of a smaller and shorter RCT (Protein, Fibre and Metabolic Syndrome; ProFiMet) demonstrated that cereal fibre may absorb branched-chain amino acids (BCAA) [17]. These particular amino acids are involved in the activation of the mammalian target of rapamycin (mTOR) and, therefore, the promotion of insulin resistance. In ProFiMet, the detected metabolic improvement in insulin action by fibre intake was limited to a period of 6 weeks, while there was no residual middle-term benefit after 18 weeks [17].

Both OptiFiT and ProFiMet might have failed to show stronger results concerning fibre effects due to their specific cohort structure. OptiFiT selectively recruited subjects with impaired glucose tolerance (IGT), a prediabetes condition that is more abundant among women [18]. IGT is a strong predictor of diabetes onset within the next few years and mainly represents failure of postprandial insulin secretion and/or peripheral insulin resistance. In contrast, impaired fasting glucose (IFG) is more tightly linked to hepatic insulin resistance and nonalcoholic fatty liver disease (NAFLD). ProFiMet recruited only women with metabolic syndrome who did not require more specific, mandatory metabolic alterations (such as prediabetes, NAFLD or obesity).

In 2018, several working groups demonstrated that T2DM is a heterogenous metabolic disorder. Two Scandinavian cohort studies on new-onset T2DM patients described phenotype clusters, separating groups of patients with different long-term outcomes regarding metabolic state and complications. Both studies used measures of obesity to distinguish patient clusters [19,20].

Based on such stratification techniques, intervention studies, too, need to be reassessed. It is expected that these clusters of T2DM phenotypes not only differ in their pathophysiological backgrounds but, in fact, also respond differently to dietary or pharmacological interventions. For example, there is evidence that fasting glucose levels affect the metabolic response to hypocaloric or fibre-rich diets [21,22].

The first stratification approach of OptiFiT replicated the last-mentioned findings: subjects with IFG showed a stronger metabolic benefit from fibre supplementation, while participants with normal fasting glucose (NFG) seemed to achieve metabolic amelioration predominantly on the basis of general lifestyle adaptation rather than fibre supplementation [23]. IFG and fasting insulin resistance rather than IGT and dynamic insulin resistance are strongly associated with hepatic insulin resistance and NAFLD [18,24,25]. As NFG and IFG subjects not only differ in terms of fasting glucose levels but also body mass index (BMI), it is unclear, if either glycemic fasting state (mirroring liver fat content) or surplus of (visceral) adipose tissue is the relevant modulating factor. A separate analysis is necessary, as there is no full overlap between IFG and obesity or NFG and absence of obesity.

We therefore decided to perform a second stratified analysis of OptiFiT based on the pretreatment presence of obesity to investigate the hypothesis that glycometabolic benefits of insoluble cereal fibre are modulated by the state of obesity.

## 2. Research Design and Methods

Recruitment, inclusion and exclusion criteria and overall study design have been published elsewhere [15]. OptiFiT is a randomised controlled double-blinded intervention study, which recruited 180 subjects with IGT in order to recruit subjects with an increased risk for diabetes onset. The study protocol was registered at clinicaltrials.gov: NCT 01681173.

Most of the subjects fulfilled the definition of metabolic syndrome. One hundred and thirty-six subjects completed the first year of intervention and, thus, formed the per-protocol dataset.

For the presented work, the cohort was stratified by baseline obesity state, resulting in 87 (63%) subjects with obesity (OBESE) and 49 (37%) with a BMI below 30 kg/m² (NONOBESE).

All 180 participants underwent the 1 year structured “Treatment and Education Program for Prevention of Type 2 Diabetes” (PREDIAS) [26,27]. Based on the PREDIAS framework, we defined specific dietary goals that were in accordance with the recommendations of the German Nutrition Society (Deutsche Gesellschaft für Ernährung; DGE): fat intake below 30 kcal%, intake of saturated fat below 10 kcal% and intake of dietary fibre above 15 g/1000 kcal. We recommended frequent ingestion of whole-grain products, legumes, vegetables, fruits (in particular, berries), low-fat milk and meat products, soft margarines and vegetable oils rich in unsaturated fatty acids [15]. We encouraged physical activity (PA; 240 min/week) and used pedometers and the European Physical Activity Questionnaire (EPAQ-2) in order to monitor physical activity [15,28].

Compliance to dietary advice was monitored by 4-day food records every 6 months. Nutrient intake was determined using the nutrition software PRODI^®^ 5.8 (Nutri-Science, Hausach, Baden-Wuerttemberg, Germany) based on Bundeslebensmittelschlüssel 3.0 [29].

To assess metabolic state, oGTTs (75 g of glucose in 300 mL of water; AccuChek Dextrose O.G.T.) with blood sampling for levels of glucose, insulin and C-peptide were used. Samples were taken every 30 min. Fasting glucose, 2 h glucose levels and HbA1c were defined as primary outcome variables. A wide panel of additional fasting blood parameters was measured to evaluate inflammation, lipid levels and liver function.

### 2.1. Dietary Supplement

Details on the supplementation procedure, measurements and laboratory parameters have been given elsewhere [15]. The participants were provided with drinking powder supplements. The fibre supplement contained a purified fibre extract derived from oat hulls (70 w (weight) % cellulose, 25 w% hemicellulose and 3–5 w% lignin (Vitacel OF 560-30; Rettenmaier & Söhne, Holzmuehle, Germany)). Placebo consisted of waxy maize starch with a negligible content of insoluble fibre and guar gum and isomaltulose. Our subjects were asked to consume the supplements twice a day after dissolving the recommended amount of drinking powder (two large 10 mL scoops provided with the supplement tins) in 300 mL of water. By doing so, subjects in the fibre group achieved an additional daily intake of 15 g of mainly insoluble fibre on top of their regular diet. Both supplements were similar in appearance, taste, odour and texture. Volunteers were randomly assigned using a computer algorithm to either placebo or fibre supplement by office staff who were not involved in other study procedures. The randomised supplement allocation was blinded to both participants and study personnel. Supplement tins were weighed accurately before distribution and weighed again when returned after use at the main visits [15].

### 2.2. Calculations

Areas under the curve (AUC) for oGTT responses (plasma glucose, insulin and C-peptide) were calculated by the trapezoidal method. We assessed the homeostasis model assessment index for insulin resistance (HOMA_IR_) [30]; the quantitative insulin sensitivity checks index (QUICKI), the insulin sensitivity index, including levels for free fatty acids (ISI_ffa_) [31]; dynamic insulin sensitivity indices by Cederholm and Belfiore [32,33]; the fatty liver index (FLI) [34] as well as hepatic insulin clearance.

### 2.3. Statistical Analyses

We used the Kolmogorov–Smirnov test in order to determine normal distribution of our data. Given the frequent absence of normal distribution, we decided to conduct nonparametric tests for the entire trial to ensure uniform representation, namely, Mann–Whitney tests for cross-sectional comparisons and Wilcoxon tests for longitudinal comparisons. In case of significant differences between groups, a three-way ANOVA (mixed linear model; time × diet × obesity baseline status; adjusted for weight change) was conducted. For nonprimary outcomes, a Benjamini–Hochberg correction for multiple testing was performed (FDR 0.05; *n* = 20). All data are presented as mean ± standard deviation. The results were considered significantly different if *p* < 0.05. While the original full dataset was planned to achieve a power of 80%, comparison of only OBESE subjects provided a power of 35%, assuming the same effect sizes [14]. All statistical analyses were performed using SPSS for Windows program version 22.0 (SPSS Inc., Chicago, IL, USA).

## 3. Results

Baseline conditions are presented in Table 1 for OBESE and NONOBESE subjects. There were no significant differences between the fibre groups compared to their respective placebo counterparts, except for sex ratio between the two OBESE subgroups. As expected, OBESE subjects showed significantly higher values for all anthropometric variables but also fasting insulin and fasting C-peptide, as well as measures of insulin resistance, hepatic insulin clearance (HIC), C-reactive protein (CRP) and leukocyte count, when compared with NONOBESE subjects. 

Dietary intake of calories, macronutrients and fibre from conventional food did not differ between the four subgroups at baseline. The intake of calories and total fat decreased significantly within all subgroups, except for the OBESE fibre group after 1 year of intervention. However, every subgroup surpassed the aimed threshold of 30% of energy intake by dietary fat. Only a minority of all subjects managed to achieve a low-fat intake according to the recommendations. Alcohol intake decreased significantly in all but the NONOBESE placebo group, whereas protein intake decreased in the OBESE fibre group only. There were no statistical differences between the groups when comparing dietary changes. Physical activity did not change within any of the subgroups and there were no statistically significant differences between the groups concerning change in PA (Table 2).

Major glycemic outcomes (fasting glucose, 2 h glucose and HbA1c) did not change in a statistically significant way in any of the four subgroups, except for 2 h glucose in the OBESE fibre group, and again, there were no relevant differences between the groups (Table 3).

Between the two NONOBESE groups (fibre: *n* = 26; placebo: *n* = 23), there was no significant difference in the change of anthropometric or metabolic parameters. Within the NONOBESE fibre group, significant improvements were detected for body weight as well as waist and hip circumferences. Within the NONOBESE placebo group, additional improvements were seen with respect to fasting insulin, HOMA_IR_, QUICKI, Belfiore index and HIC (Table 3).

Between the two OBESE intervention groups (fibre: *n* = 41; placebo: *n* = 46), there was a significant difference in the change of leukocyte count and gamma-glutamyl transferase (GGT) levels during the 12-month intervention, but only the first effect survived correction for multiple testing (*p*-value < 0.001; corrected level of significance α = 0.0025). Both the OBESE fibre group and the OBESE placebo group experienced a significant reduction of body weight, waist and hip circumferences as well as fasting (QUICKI) and postprandial insulin resistance (Belfiore index) (Table 3).

Among the placebo groups, NONOBESE subjects had a stronger reduction in body fat content compared with OBESE subjects. OBESE subjects allocated to fibre supplementation experienced a significantly better improvement of fasting C-peptide and leukocyte count compared with their NONOBESE counterparts. This effect on leukocyte count did not survive Benjamini–Hochberg correction for multiple testing (*p*-value of 0.006; corrected level of significance α = 0.0025) (Table 3).

Overall, we found a significant interaction effect for leukocyte count (time × supplement × obesity status), indicating that improvement of leukocyte count was strongest in OBESE subjects receiving the fibre supplement, as shown in the pairwise comparisons.

## 4. Discussion

In this secondary post hoc analysis, improvements in major glycemic outcomes were only achieved in one of the four stratified intervention arms (OBESE fibre). Numerically, both fibre arms had a small benefit in 2 h glucose levels, which is completely absent in the placebo group. Stratification led to lower power compared with the original study and, thus, failed to replicate the results from the original publication of OptiFiT [15]. On the other hand, stratification by IFG state created subgroups of similar group size and without significant baseline differences between the respective fibre and placebo counterparts. This approach demonstrated a surplus benefit of cereal fibre in subjects with combined IFG/IGT compared with patients with isolated IGT. Our current analysis seems to underline that the “fibre effect” is indeed driven by the glycometabolic state itself rather than concomitant differences in body weight between IFG and NFG strata [23].

The current study shows that the modified PREDIAS program was successful in actively promoting a healthier lifestyle in both OBESE and NONOBESE subjects, mainly by reducing fat intake and thus total energy intake. This explains weight loss in all four subgroups and possible concomitant improvements in metabolic outcomes. Decreased intake of saturated fat can support the reduction of inflammatory processes (e.g., in visceral fat depots [35,36,37]). Advice for increased fibre load by the daily diet has turned out to be mostly fruitless in the OptiFiT subjects, highlighting the need for fibre supplementation or fortified food products [38]. Similarly, increase of PA is often recommended but rarely set in motion for a long period of time [39]. In all four subgroups, subjects failed to increase their daily energy expenditure by increasing walking distance in a relevant magnitude, and no group surpassed one of the others in its (minuscule) progress.

In this stratified approach, the statistical power of demonstrating a tight relation between obesity state and glycometabolic improvement was limited. Yet, compliance with low-fat regimens is also often limited as well [40,41].

Surprisingly, there are still significant differences between some of the investigated subgroups. First of all, leukocyte count improved significantly more within the OBESE fibre group than in its NONOBESE counterpart. This was true, although the OBESE subgroup did not experience a significant reduction of saturated fat intake or weight loss, ruling out a relevant contribution to this effect. Therefore, this finding might instead be explained by fibre supplementation. The original paper already reported a significant benefit of fibre supplementation on leukocyte count, and there are several cohort studies supporting anti-inflammatory effects [42,43,44,45]. There is no known way of action, especially as our cereal fibre is hardly fermentable and thus biologically inert. There is a need for confirmatory and mechanistic studies, investigating interactions of insoluble fibres with the gut microbiome beyond the limited potential of SCFA production.

The described benefit of the OBESE fibre group with respect to leukocyte count also needs to be interpreted in comparison to the NONOBESE fibre group. Possibly, only OBESE subjects provided a sufficiently elevated inflammatory level that allowed for amelioration, while NONOBESE subjects presented a bottom effect.

A similar difference can be seen between the OBESE and NONOBESE placebo groups, with the OBESE one managing a greater loss of body fat. Once again, this result seems to be driven mainly by baseline levels.

More interesting from a mechanistic perspective is the significant improvement in GGT levels, which was only present within the OBESE fibre group and was significantly greater when compared with placebo. This might indicate that the metabolic benefits of cereal fibre do not depend on visceral fat but hepatic steatosis. As there are almost no previous data on the effects of cereal fibre on NAFLD in humans or animals, we can only speculate about an underlying possible, plausible explanation. Rodent trials on fibre and NAFLD reported concomitant improvements in serum triglyceride and cholesterol levels, which we did not see [46,47,48]. Given the heterogeneous nature of T2DM and its “precursor”, prediabetes, certain subphenotypes might benefit from cereal fibre in a more extensive manner than others. Subjects with NAFLD often show (hepatic) insulin resistance with compensatory hyperinsulinemia. If cereal fibre specifically counteracts these crucial pathways, benefits might be limited in unaffected subjects, although nevertheless presenting similar dysglycemia. Our previous analysis on IFG as a modulating factor for fibre effects in OpTiFiT has confirmed findings from other recent trials, which reported a dependence of metabolic amelioration on pretreatment glycemic state [18,24,25].

This would also explain the significant difference between OBESE and NONOBESE subjects in the fibre arm: fasting C-peptide levels showed a small increase in the NONOBESE subgroup and stable-to-dropping levels within the OBESE subgroup. Improved insulin sensitivity should be accommodated by a reduction of insulin secretion. This would especially be expected in subjects with obesity and/or NAFLD, who show signs of compensatory hyperinsulinemia. Increasing insulin secretion (represented by increasing C-peptide levels) in response to an antidiabetic treatment might indicate two mechanisms: stronger compensation of growing insulin resistance or restoration of initially impaired insulin secretion capacity. Primary insulin deficiency is a common trait in patients with T2DM, especially in patients who are not obese. About one-third of all T2DM patients have a BMI below 30 kg/m². Despite having low amounts of visceral and hepatic fat, these patients are at high risk for long-term complications [19,20,49]. Therefore, nonobese T2DM patients can actually be at higher risk for metabolic deterioration and mortality. Obese subjects might show secondary beta-cell failure but only after a long period of T2DM manifestation. Well-powered RCTs are required to demonstrate that established therapies for T2DM are in fact helpful for all major subtypes of this disorder. A more individualised therapeutic scheme can be expected to be established in the near future.

We are aware of certain limitations of our analysis. Stratification always leads to relatively small subgroups. OptiFiT, as typical nutritional RCT, has also a slightly imbalanced sex ratio with male subjects being under-represented.

Still, by using a double-blinded supplementation and a group-based intervention program, we were able to “homogenise” our intervention elements; all subjects received the same amount and the same content of information from the same consultants. OBESE subjects did not receive different dietary advice than NONOBESE subjects, except for goals of total energy intake.

There is no viable biomarker for the assessment of fibre intake or any major nutrient group. Food records are prone to under-reporting, and drug accounting still depends on trust and honesty of the patients. Still, our consistency analysis showed no relevant flaws, imbalances or missing correlations between food intake and body weight (development). We are convinced that all available measures for the assessment of compliance were used up to their full potential. Also, the use of pedometers and EPAQ-2 questionnaires provided additional information on a healthy lifestyle. We experienced a moderate 1-year drop-out rate of 24%, not explained by cases of incident diabetes.

For ethical reasons, we added no randomised control group without any kind of intervention. Several prevention trials have shown the potential to reduce T2DM incidence; therefore, actively avoiding lifestyle treatment for patients with confirmed IGT is not acceptable [6,7,8,9].

In summary, we conclude that obesity is not a strong modulating factor of metabolic benefits due to supplementation with cereal fibre. Surplus benefits in subjects with elevated fasting glucose (and perhaps NAFLD) are linked to their metabolic state rather than to body weight. Weight loss, as seen in our placebo groups, contributes separately to an improvement of risk factors. Cereal fibre supplementation has the added value of acting independent of obesity, weight loss and change in food choice. Therefore, improving diet during the production process before reaching the customer (i.e., by supplementation or fortification) might serve as a helpful tool for fighting the diabetes epidemic.

## Figures and Tables

**Table 1:** Characteristics of participants at study entry.

	NONOBESE Fibre	NONOBESE Placebo	OBESE Fibre	OBESE Placebo
Sex (w/m)	16/10 (62%)	11/12 (48%)	32/9 (78%)	25/21 (54%) *
Age (years)	62.0 ± 9.7	62.4 ± 9.1	58.8 ± 8.9	58.7 ± 9.1
BMI (kg/m^2^)	26.9 ± 3.2	27.3 ± 2.5	34.8 ± 3.5	36.5 ± 5.8
Weight (kg)	77.5 ± 12.0	75.9 ± 12.1	94.0 ± 14.0	103.0 ± 18.2
Waist circumference (cm)	93.8 ± 9.0	94.0 ± 9.2	107.7 ± 11.7	113.2 ± 12.3
Hip circumference (cm)	102.8 ± 8.4	102.9 ± 5.8	116.6 ± 11.3	120.2 ± 12.7
Waist-to-hip ratio (WHR)	0.91 ± 0.08	0.91 ± 0.07	0.93 ± 0.08	0.95 ± 0.09
BIA—body fat (%)	32.3 ± 9.0	31.7 ± 5.5	39.4 ± 7.0	37.9 ± 8.5
RR syst. (mmHg)	139 ± 20	140 ± 17	140 ± 16	142 ± 16
Fasting glucose (mg/dL)	89.1 ± 10.7	90.0 ± 10.2	90.7 ± 10.2	92.2 ± 10.0
2 h glucose (mg/dL)	159.5 ± 18.4	155.7 ± 16.8	156.7 ± 15.1	164.0 ± 19.4
HbA_1c_ (%)	5.5 ± 0.4	5.6 ± 0.3	5.7 ± 0.3	5.6 ± 0.4
Fasting insulin (mU/L)	6.8 ± 3.3	7.8 ± 4.5	10.3 ± 4.7	10.8 ± 5.5
Fasting C-peptide (µg/L)	1.3 ± 0.8	1.4 ± 0.8	1.8 ± 0.7	1.8 ± 0.6
HOMA_IR_	1.9 ± 1.0	2.1 ± 1.3	2.7 ± 1.4	2.9 ± 1.8
QUICKI	0.36 ± 0.03	0.36 ± 0.05	0.34 ± 0.03	0.33 ± 0.02
ISI_ffa_	1.00 ± 0.21	0.96 ± 0.36	0.78 ± 0.30	0.75 ± 0.22
Belfiore	0.79 ± 0.27	0.77 ± 0.35	0.57 ± 0.24	0.61 ± 0.23
HIC_c-peptide_ (mU/µg)	5.4 ± 2.0	5.5 ± 2.2	4.6 ± 1.5	4.7 ± 1.8
HDL cholesterol (mmol/L)	1.3 ± 0.3	1.4 ± 0.4	1.2 ± 0.2	1.2 ± 0.3
LDL cholesterol (mmol/L)	3.7 ± 0.8	3.5 ± 0.6	3.7 ± 1.0	3.5 ± 0.8
CRP (mg/L)	2.1 ± 2.4	1.2 ± 1.1	5.9 ± 5.3	4.0 ± 4.0
Leukocyte count (Gpt/L)	4.98 ± 1.00	5.12 ± 1.62	6.32 ± 1.60	5.61 ± 1.26
Uric acid (µmol/L)	348 ± 83	337 ± 93	335 ± 70	355 ± 75
GGT (U/L)	28 ± 22	31 ± 32	39 ± 40	34 ± 29
Fatty liver index (FLI)	43 ± 23	42 ± 25	83 ± 12	85 ± 14

Characteristics of NONOBESE and OBESE participants at study entry. BMI: body mass index; BIA: bioelectrical impedance analysis; CRP: C-reactive protein, GGT: gamma-glutamyl transferase; HIC: hepatic insulin clearance; HOMA_IR_: homeostatic model assessment for insulin resistance; ISI: insulin sensitivity index; QUICKI: quantitative insulin sensitivity check index; RR: blood pressure (Riva-Rocci). * Significant difference between the respective fibre and placebo subgroup, X^2^ test or Mann–Whitney *U* test; * *p* < 0.

**Table 2:** Lifestyle habits at baseline and after 1 year of intervention.

	NONOBESE Fibre (Baseline)	NONOBESE Placebo (Baseline)	OBESE Fibre (Baseline)	OBESE Placebo (Baseline)	NONOBESE Fibre (12 Months)	NONOBESE Placebo (12 Months)	OBESE Fibre (12 Months)	OBESE Placebo (12 Months)
Food intake								
Total energy intake (kcal/day)	2101 ± 519	1938 ± 515	2015 ± 443	2042 ± 611	1767 ± 450 *	1830 ± 489 *	1718 ± 349	1953 ± 569 *
Fat intake (g/day)	82 ± 24	73 ± 22	78 ± 25	81 ± 31	66 ± 21 *	65 ± 23 *	66 ± 22 *	75 ± 31
Saturated fat (g/day)	35 ± 13	31 ± 11	34 ± 11	36 ± 13	27 ± 11 *	27 ± 10 *	28 ± 10 **	33 ± 13
Fat intake (kcal%)	37 ± 6	36 ± 5	36 ± 7	37 ± 6	34 ± 6	32 ± 5	35 ± 7	35 ± 6
Protein intake (g/day)	80 ± 19	77 ± 18	83 ± 26	86 ± 24	73 ± 22	74 ± 21	70 ± 19 **	84 ± 22
Carbohydrate intake (g/day)	234 ± 66	218 ± 59	222 ± 53	224 ± 76	205 ± 52	214 ± 51	201 ± 40	220 ± 59
Total dietary fibre intake (g/day)	24 ± 6	24 ± 6	22 ± 7	23 ± 8	24 ± 6	26 ± 10	22 ± 7	24 ± 8
Insoluble	16 ± 4	16 ± 4	15 ± 5	15 ± 5	14 ± 5	16 ± 5	15 ± 6	15 ± 5
Soluble	7 ± 2	7 ± 2	7 ± 2	7 ± 2	7 ± 2	7 ± 2	7 ± 2	7 ± 2
Alcohol (g/day)	10 ± 12	11 ± 14	11 ± 18	7 ± 13	6 ± 8 *	10 ± 16	5 ± 8 *	5 ± 8 *
Physical activity								
Steps per day (*n*)	7381 ± 3200	5921 ± 1873	6255 ± 2797	6442 ± 2873	6964 ± 2651	6434 ± 3816	7769 ± 3608	6988 ± 3184
Energy expenditure by steps (kcal/day)	492 ± 271	398 ± 159	432 ± 205	449 ± 256	426 ± 150	421 ± 241	515 ± 234	471 ± 218

Changes in lifestyle habits during intervention; data are mean ± SD. Nutrient intakes were calculated from 4-day food records. Physical activity was derived from 1-week assessments with pedometers. * Significant change from baseline to 12-month follow-up within the respective subgroup, Wilcoxon tests, * *p* < 0.05, ** *p* < 0.001.

**Table 3:** Changes in anthropometric and metabolic parameters after 12 months.

	NONOBESE Fibre	NONOBESE Placebo	OBESE Fibre	OBESE Placebo	NONOBESE: Fibre vs. Placebo	OBESE: Fibre vs. Placebo	Placebo: NONOBESE vs. OBESE	Fibre: NONOBESE vs. OBESE
Weight (kg)	−2.4 ± 3.2 **	−2.0 ± 3.2 **	−2.8 ± 5.3 **	−3.6 ± 6.6 ***	0.554	0.521	0.265	0.877
Waist circumference (cm)	−2.6 ± 3.7 **	−2.9 ± 5.6 *	−3.1 ± 5.9 **	−3.5 ± 7.2 **	0.353	0.720	0.410	0.556
Hip circumference (cm)	−2.3 ± 4.4 *	−1.7 ± 2.6 **	−2.4 ± 4.5 **	−3.2 ± 6.2 **	0.877	0.622	0.484	0.995
Waist-to-hip ratio (WHR)	−0.01 ± 0.04	−0.01 ± 0.04	−0.01 ± 0.04	−0.00 ± 0.06	0.516	0.772	0.712	0.786
BIA—body fat (%)	0.2 ± 5.4	−2.7 ± 6.4	−0.9 ± 4.2	−0.3 ± 3.3	0.096	0.502	0.040 †	0.293
RR syst. (mmHg)	−7 ± 15	1 ± 17	0 ± 16	−3 ± 18	0.211	0.488	0.576	0.214
Fasting glucose (mg/dL)	−0.4 ± 11.5	−0.6 ± 7.7	−2.8 ± 10.7	−1.7 ± 8.9	0.944	0.524	0.819	0.358
2 h glucose (mg/dL)	−8.4 ± 34.8	0.1 ± 31.7	−12.9 ± 25.2 **	−6.7 ± 30.4	0.214	0.356	0.449	0.827
HbA_1c_ (%)	0.1 ± 0.5	0.1 ± 0.5	−0.1 ± 0.4	0.1 ± 0.5	0.767	0.052	0.679	0.072
Fasting insulin (mU/L)	−0.9 ± 2.2	−1.8 ± 2.9 *	−1.9 ± 3.9 **	−0.7 ± 5.5	0.268	0.304	0.681	0.094
Fasting C-peptide (µg/L)	0.4 ± 1.6	0.2 ± 1.3	−0.1 ± 1.0	0.5 ± 1.6	0.250	0.046	0.627	0.035 †
HOMA_IR_	−0.3 ± 0.7	−0.5 ± 0.8 **	−0.6 ± 1.2 **	−0.3 ± 1.6	0.307	0.361	0.701	0.146
QUICKI	0.01 ± 0.03	0.02 ± 0.03 *	0.01 ± 0.03 *	0.01 ± 0.03 *	0.360	0.493	0.350	0.567
ISI_ffa_	0.03 ± 0.23	0.11 ± 0.35	0.09 ± 0.30	0.08 ± 0.31	0.287	0.434	0.480	0.314
Belfiore	0.07 ± 0.37	0.11 ± 0.23 *	0.17 ± 0.22 **	0.14 ± 0.27 **	0.939	0.651	0.558	0.421
HIC_c-peptide_ (mU/µg)	1.1 ± 3.0	0.9 ± 1.6 *	1.3 ± 2.0 **	1.9 ± 2.7 *	0.851	0.331	0.222	0.556
HDL cholesterol (mmol/L)	0.0 ± 0.2	0.0 ± 0.3	0.0 ± 0.1	−0.0 ± 0.2	0.833	0.204	0.780	0.571
LDL cholesterol (mmol/L)	−0.2 ± 1.1	−0.1 ± 0.8	−0.0 ± 0.5	0.1 ± 0.9	0.075	0.570	0.441	0.106
CRP (mg/L)	−0.4 ± 2.2	−0.1 ± 1.3	−1.6 ± 4.4 *	−0.8 ± 3.2	0.928	0.904	0.098	0.129
Leukocyte count (Gpt/L)	0.11 ± 1.09	−0.30 ± 1.07	−0.95 ± 1.26 **	0.26 ± 0.96	0.239	**<0.001 †††**	0.072	0.006 ††
Uric acid (µmol/L)	−22 ± 56	−18 ± 55	−4 ± 59	−3 ± 59	0.865	0.993	0.257	0.157
GGT (U/L)	−2 ± 11	−1 ± 17	−9 ± 37 *	2 ± 26	0.173	0.042 †	0.577	0.203
FLI	−5 ± 14	−5 ± 13	−7 ± 15 *	−4 ± 13	0.926	0.409	0.741	0.797

Outcomes during intervention; mean ± SD. * Significant change within the group; Wilcoxon tests; * *p* < 0.05, ** *p* < 0.01, *** *p* < 0.001.; † Significant difference between groups; Mann–Whitney *U* test; † *p* < 0.05, †† *p* < 0.01, ††† *p* < 0.001. *p*-values in bold print have survived Benjamini–Hochberg correction for multiple testing. BMI: body mass index; BIA: bioelectrical impedance analysis; CRP: C-reactive protein, GGT: gamma-glutamyl transferase; HIC: hepatic insulin clearance; HOMA_IR_: homeostatic model assessment for insulin resistance; ISI: insulin sensitivity index; QUICKI: quantitative insulin sensitivity check index; RR: blood pressure (Riva-Rocci).

## Data Availability

Datasets are available by request to the corresponding author.

## Abbreviations

ALAT	alanine-amino transferase
ASAT	aspartate-amino transferase
AUC	area under the curve
BCAA	branched-chain amino acid
BIA	bioelectric impedance analysis
CRP	C-reactive protein
EPAQ	European Physical Activity Questionnaire
FLI	fatty liver index
GGT	gamma-glutamyl transferase
GLP-1	glucagon-like peptide 1
HbA1c	glycated haemoglobin
HDL	high-density lipoprotein
HIC	hepatic insulin clearance
HOMA_IR_	homeostasis model assessment insulin resistance index
IFG	impaired fasting glucose
IGT	impaired glucose tolerance
ISI_ffa_	insulin sensitivity index of blood-free fatty acids
LDL	low-density lipoprotein
MR-S	magnetic resonance spectroscopy
NAFLD	nonalcoholic fatty liver disease
NFG	normal fasting glucose
OptiFiT	Optimal Fibre Trial for Diabetes Prevention
OGTT	oral glucose tolerance test
PA	physical activity
PREDIAS	Prevention of Diabetes Self-Management
ProFiMet	Protein, Fibre and Metabolic Syndrome
QUICKI	quantitative insulin sensitivity check index
T2DM	type 2 diabetes mellitus
